# Reduced total energy expenditure and physical activity in cachectic patients with pancreatic cancer can be modulated by an energy and protein dense oral supplement enriched with n-3 fatty acids

**DOI:** 10.1038/sj.bjc.6601620

**Published:** 2004-03-02

**Authors:** A W G Moses, C Slater, T Preston, M D Barber, K C H Fearon

**Affiliations:** 1 Department of Clinical and Surgical Sciences (Surgery), University of Edinburgh, Royal Infirmary, Little France Crescent, Edinburgh EH16 4SA, UK; 2 Division of Developmental Medicine, University of Glasgow, Yorkhill Hospitals, Glasgow G3 8SJ, UK; 3 Scottish Universities Environmental Research, Scottish Enterprise Technology Park, Rankine Avenue, East Kilbride, Glasgow G75 0QF, UK

**Keywords:** cachexia, quality of life, energy metabolism, eicosapentaenoic acid

## Abstract

The aim of the study was to assess the total energy expenditure (TEE), resting energy expenditure (REE) and physical activity level (PAL) in home-living cachectic patients with advanced pancreatic cancer. The influence of an energy and protein dense oral supplement either enriched with or without the n-3 fatty acid eicosapentaenoic acid (EPA) and administered over an 8-week period was also determined. In total, 24 patients were studied at baseline. The total energy expenditure was measured using doubly labelled water and REE determined by indirect calorimetry. Patients were studied at baseline and then randomised to either oral nutritional supplement. Measurements were repeated at 8 weeks. At baseline, REE was increased compared with predicted values for healthy individuals (1387(42) *vs* 1268(32) kcal day^−1^, *P*=0.001), but TEE (1732(82) *vs* 1903(48) kcal day^−1^, *P*=0.023) and PAL (1.24(0.04) *vs* 1.50) were reduced. After 8 weeks, the REE, TEE and PAL of patients who received the control supplement did not change significantly. In contrast, although REE did not change, TEE and PAL increased significantly in those who received the n-3 (EPA) enriched supplement. In summary, patients with advanced pancreatic cancer were hypermetabolic. However, TEE was reduced and this was secondary to a reduction in physical activity. The control energy and protein dense oral supplement did not influence the physical activity component of TEE. In contrast, administration of the supplement enriched with EPA was associated with an increase in physical activity, which may reflect improved quality of life.

Weight loss in cancer is frequently ascribed to a combination of reduced food intake and hypermetabolism. However, the metabolic response to cancer is heterogeneous. Some patients show an increase in resting energy expenditure (REE), while others show either no change or are hypometabolic (Dempsey *et al*, 1986). It has been suggested that in cachectic patients with lung cancer, although REE may be elevated, total energy expenditure (TEE) is decreased (Gibney *et al*, 1997). Apart from REE, the major contributor to TEE is the energy associated with physical activity. Thus, weight-losing cancer patients appear to reduce the magnitude of their evident energy deficit by a reduction in physical activity. Clearly, such a phenomenon will impact on their quality of life. We have demonstrated previously that patients with advanced pancreatic cancer have an increased REE (Falconer *et al*, 1994). However, the effects of pancreatic cancer on TEE and physical activity level (PAL) are unknown.

The mediators of anorexia and metabolic change in cancer patients are thought to include proinflammatory cytokines, neuroendocrine stress hormones and tumour-specific cachectic factors such as proteolysis-inducing factor (PIF) (Tisdale, 2002). Eicosapentaenoic acid (EPA) is an n-3 fatty acid, which has been shown to downregulate proinflammatory cytokine release both in healthy individuals (Endres *et al*, 1989) and in cachectic cancer patients (Wigmore *et al* 1997; Moses *et al*, 2002). Eicosapentaenoic acid has also been shown to inhibit muscle protein catabolism induced by PIF (Lorite *et al*, 1997) by inhibiting the activation of the ubiquitin proteasome pathway (Whitehouse *et al*, 2001). The administration of EPA either as fish oil capsules (Wigmore *et al*, 1996) or in a purified form (Wigmore *et al*, 2000) to cachectic cancer patients has been associated with weight stabilisation. Eicosapentaenoic acid combined with an energy and protein dense oral supplement has been shown to be associated with increased lean body mass (LBM), normalisation of elevated REE and improved performance status (Barber *et al*, 1999).

The present study was part of a randomised controlled trial comparing the effects of an EPA containing nutritional supplement with those of an isocaloric, isonitrogenous control supplement on weight, LBM and quality of life in patients with pancreatic cancer cachexia (Fearon *et al*, 2003). The aim of this particular study was to determine whether the decreased TEE and PAL observed in lung cancer patients (Gibney *et al*, 1997) is also observed in patients with gastrointestinal (pancreatic) malignancy and to test the hypothesis that the combination of EPA with an energy and protein dense supplement might be associated with stabilisation or gain in weight and improved TEE/PAL as a potential objective measure of quality of life.

## PATIENTS AND METHODS

### Patients

A total of 24 patients with unresectable pancreatic cancer who had been referred to the Royal Infirmary of Edinburgh were included in the study if they had lost more than 5% of their preillness stable weight over the previous 6 months, had a Karnofsky performance score of 60 or more and had a life expectancy greater than 2 months. Patients' average survival was, in fact, 130 days from the time of recruitment. All patients underwent staging by CT scan. In all, 17 patients had histological proof of diagnosis and seven patients were diagnosed on the basis of unequivocal clinical (operative) or radiological (CT scan) findings. In total, 16 patients underwent laparoscopy and 13 patients underwent laparotomy. Patients with pancreatic cancer were selected for this study as these patients usually experience severe weight loss associated with cancer cachexia. Patients were excluded if they had: undergone major surgery, endoscopic stenting, radiotherapy or chemotherapy during the previous 4 weeks; other active medical conditions (e.g. major gastrointestinal disease, chronic renal failure, uncontrolled diabetes and human immunodeficiency virus); a body mass index (BMI) greater than 30 kg m^−2^; received medication that could profoundly modulate metabolism or weight, in particular, the use of fish oil or n-3 fatty acid preparations exceeding 200 mg day^−1^ EPA or one capsule of fish oil/day within the previous 90 days. At the time of enrolment no patient had jaundice, pyrexia, severe anaemia, clinical or radiological evidence of infection and none were taking steroids at doses above that for physiological replacement. No patients had ascites or dependent ankle oedema. Pancreatic enzyme supplements were administered if patients had or developed clinical evidence of steatorrhoea. The Lothian Region Ethics Committee for human research approved the protocol, and written informed consent was obtained from all patients. Procedures followed were in accordance with the Helsinki Declaration of 1975, as revised in 1983.

#### Study design

The study was undertaken at one centre (Royal Infirmary, Edinburgh) on patients who had also been included in a multicentre, randomised, double-blind trial (Fearon *et al*, 2003). Patients were asked to consume two cans per day of either an n-3 fatty acid containing oral nutritional supplement or an identical supplement without n-3 fatty acids for an 8-week period. Both oral supplements were provided by Ross Products Division, Abbott Laboratories, Columbus, OH, USA and were ready-to-use, calorically dense, high-protein, low-fat formulations intended to act as a supplement to the patient's usual diet. Each 237 ml can provide 310 kcal, 16 g protein, 6 g fat, with or without 1.1 g of the n-3 fatty acid, EPA (Barber *et al*, 1999). The control and experimental (n-3 enriched) oral supplements were isocaloric and isonitrogenous. The increase in the n-3 fatty acid content of the experimental supplement was balanced by an increase in the n-9 (oleic) fatty acid content of the control supplement. Compliance was evaluated by the supplement consumption records kept daily by patients and after the blind was broken by measurement of plasma phospholipid EPA levels. Within the multicentre study, patients were randomised after stratifying for study site and histological proof of diagnosis to permutation blocks of two using a sequential series of numbered, sealed, opaque envelopes containing computer-generated random assignments. A copy of the randomisation sequence was kept in a locked cabinet not available to study personnel. Randomisation envelopes were opened by personnel shipping the product directly to the patients' homes. The patients, investigators and study personnel were blinded to the treatment group allocation. Study products were not distinguishable from each other in appearance, texture or taste. The blind was broken after all sample analyses were completed.

At enrolment into the study, the patients' weight, height and body composition using bioelectrical impedence were measured. Over the following 14 days, a doubly labelled water protocol was followed to measure TEE. A 3-day diet diary was completed during this period. On day 14 of the protocol, the patients attended the baseline visit. Resting energy expenditure was measured by indirect calorimetry. Weight was measured and body composition analysis was repeated using bioelectrical impedence analysis.

Patients consumed up to two cans per day for the next 8 weeks. At the start of the 7th week, patients underwent repeat measurement of TEE using the doubly labelled water protocol (which ran for 14 days to the end of week 8). A further 3-day diet diary was completed during this time. At the end of the 8 weeks of oral supplementation, patients returned for physical examination and measurement of weight, body composition, REE and plasma EPA levels.

### Weight and body composition

At the initial assessment, preillness stable weight and duration of weight loss were self-reported. Patients' height was measured and they were weighed on spring balance scales (Tanita Solar Powered Scale Model 1618, Tanita, Uxbridge, Middlesex, UK) without shoes and wearing light clothing. The same scales were used for consecutive visits.

Body composition for isotope dosing was assessed using a Xitron Hydra multiple-frequency bioelectrical impedance analyser (Xitron Technologies, San Diego, California, USA) as previously described (Hannan *et al*, 1995). Resistance was measured at 5 and 200 kHz. Total body water (TBW) was derived using equations validated in a similar patient group (Hannan *et al*, 1995). Lean body mass was calculated assuming that lean tissue contains 73% water.

### Resting energy expenditure

Following an overnight fast, patients attended at 0800. Patients rested in a supine position for at least 30 min before undergoing indirect calorimetry using a ventilated hood technique (Deltatrac; Datex, Helsinki, Finland) (Falconer *et al*, 1994). This system provides measurements of VO_2_ and VCO_2_, which have an error of less than 4% (Makita *et al*, 1990). Measurements were performed for at least 30 min. The measurements performed in the last 20 min were averaged to calculate REE using the Weir (1949) equation. Predicted values for REE were derived from the equations of Schofield (Schofield, 1985).

### Preparation of doubly labelled water

At enrolment, patient's TBW was derived from the measurements of height, weight and bioimpedance at 200 kHz (Hannan *et al*, 1995). The TBW pool was enriched with ^2^H and ^18^O to a target initial concentration of 125 parts per million excess. The weight (g) of ^2^H_2_O required (g) was calculated thus: TBW (kg) × 0.125. The weight of 10 atom% ^18^O–H_2_O (g) was calculated thus: TBW (kg) × 1.25. The ^2^H_2_O was added to a glass bottle and the weight was recorded to four decimal places. The H_2_^18^O was then added and again the weight was recorded to four decimal places. The bottle was sealed with a plastic cap and parafilm (American National Can, Menasha, WI, USA via BDH Laboratory Supplies). Prior to sealing the bottle, a 100 *μ*l aliquot was removed and added to a preweighed 100-ml glass flask containing 90 ml of tap water, which was then weighed to four decimal places. The flask was then filled with tap water to a total volume of 100 ml and then reweighed. The bottle was thoroughly mixed and a 20 ml aliquot was then added to a separate glass bottle, sealed and kept frozen at −20°C pending analysis. A sample of the tap water used for dilution was stored similarly.

### Doubly labelled water protocol

On day 0, the subject collected their second urine sample of the day and poured an aliquot into a 30 ml glass bottle and recorded the time. The doubly labelled water was then consumed by the patient. Thereafter, the bottle that had contained the labelled water was rinsed with tap water and then the contents drunk to ensure that all labelled water had been ingested. On days 1, 2, 3, 12, 13 and 14, part of the second urine sample of the day was transferred to a 30 ml glass bottle and the time was recorded. All urine samples were sent by post to the laboratory in preprepared envelopes and on receipt frozen at −20°C prior to analysis.

### Analysis of urine samples

#### ^2^H analysis

Samples were prepared according to the method of Scrimgeour *et al* (1993). Urine samples were thawed completely, shaken and centrifuged at 1000 **g** for 5 min. Samples were prepared in duplicate. Urine (400 *μ*l) was pipetted into 10 ml Exetainer gas testing vials (Labco, High Wycombe, Berks); glass inserts (200 *μ*l, Chromacol, Welwyn Garden City, Herts) containing platinum catalyst (platinum 5% on alumina powder, 325 surface area >250 m^2^ g^−1^, Sigma Aldrich, Gillingham, Dorset) were added to each vial, taking care not to wet the catalyst. Reference samples (0 and 75 ppm excess ^2^H) were prepared and analysed with each batch. Exetainer vials were placed on a 20-tube manifold and were evacuated for 1 min. The manifold was brought to atmospheric pressure prior to flushing with reference gas for 5 min. Samples were filled with reference gas (5% hydrogen in helium, Air Products Special Gases, Crewe) for 10 s and left to equilibrate at room temperature for a minimum of 48 h prior analysis. During this time the deuterium in the water phase equilibrates with the hydrogen in the gas phase. The abundance of deuterium in the gas phase was measured using a continuous flow isotope ratio mass spectrometer (CF-IRMS, Hydra, PDZ Europa, Crewe) (Prosser and Scrimgeour, 1995), which had been calibrated against international standards. The abundance of ^2^H in patient samples was calculated with reference to the known abundance of the reference samples.

#### ^18^O analysis

Samples were prepared for ^18^O analysis according to the method of Prosser *et al* (1991). After deuterium analysis, the samples were refrigerated until required for ^18^O analysis. Vials were evacuated as for deuterium analysis and filled with reference gas (3% CO_2_ in nitrogen, Air Products Special Gases, Crewe). Reference samples (0 and 150 ppm excess ^18^O) were prepared and analysed with each batch. Samples were left to equilibrate for 24 h at ambient temperature. The abundance of ^18^O in the gas phase was measured by CF-IRMS. The abundance of patients' samples was calculated with reference to the known abundance of the reference samples.

### Calculation of TEE

‘Multipoint’ calculations were used to derive turnover rates and initial enrichments of each isotope, to estimate CO_2_ production and TBW, respectively. Schoeller's equation for estimating TEE was used in the form given by Goran *et al* (1994). A resampling procedure was used to estimate the errors in (TBW) and TEE measurement (Wolfe, 1992). The precision of TBW analysis was 0.16 kg with ^18^O (s.d.) and 0.18 kg with ^2^H. TEE errors estimated by the resampling procedure averaged 4.8% (0.32 (s.d. 0.17) MJ day^−1^). Tracer elimination rate was normal (*k*_O_/*k*_H_=1.279, s.d. 0.071 and the average ^2^H : ^18^O distribution volume or pool space ratio was 1.0316 (s.d. 0.055). Predicted values for TEE were derived from predicted REE values (Schofield, 1985) multiplied by 1.5. This prediction derives from the lifestyle category defined as ‘Seated work with no option of moving around and little or no strenuous activity’ given a PAL range of 1.4–1.5 by Black *et al* (1996).

### Calculation of PAL

PAL was calculated from the formula PAL=TEE/REE. A PAL of 1.5 for healthy sedentary adults was derived from the work of Black *et al* (1996).

### Calculation of energy expenditure of activity (EEA)

EEA was calculated from the formula EEA=TEE−REE. This definition of EEA includes dietary-induced thermogenesis and nonexercise activity thermogenesis (Levine *et al*, 2001)

### Dietary intake

A 3-day diet diary completed prior to baseline (week 0), and during week 8, was used to assess the patients' dietary intake. A dietitian instructed patients on how to record food and beverage intake. The mean total energy intake (TEI) and macronutrient intakes were calculated using a computerised dietary analysis. Patients were also asked to record the number of cans of supplement, or parts thereof, consumed each day. The total dietary intake was calculated by adding oral supplement consumption to spontaneous food intake. Predicted intakes at baseline assumed to be the same as predicted TEE on the basis that this is what would be required to allow weight stability.

### Plasma fatty acid analysis

Analysis of EPA in patients' plasma phospholipids before study commencement and at 8 weeks was performed by gas chromatography as described previously (Wigmore *et al*, 1996). A plasma EPA level of 1.6% is approximately the 90th percentile in free-living unsupplemented pancreatic cancer patients (Barber *et al*, 1999; Zuijdgeest-Van Leeuwen *et al*, 2002).

### Physician-assessed physical function score

Patients were assessed for their level of physical function using the Karnofsky performance score.

### Statistical analysis

The results are expressed as mean (standard error of the mean: s.e.m.). A Student's unpaired *t*-test for independent samples was used to look for the differences between groups, while a paired Student's *t*-test was used to look for the differences within a group. Fisher's exact test and Wilcoxon's test were used where appropriate. Differences were considered significant at *P*<0.05. A sample size of 16 patients was calculated to detect a 20% difference in PAL between groups with a significance level of 0.05 and a power of 0.8. The target sample size was increased to 30 patients to account for possible attrition of patients during the study.

## RESULTS

The characteristics of the 24 patients evaluated at baseline are shown in Table 1

**Table 1:** Baseline characteristics of weight-losing patients with unresectable pancreatic cancer (*n*=24)

**Baseline characteristics**	**Patients (*n*=24)**
Sex (M : F)	10 : 14
Age (years)	68 (2)
*Stage of disease*	
II	15
III	4
IV	5

BMI	20 (1)
% weight loss from usual weight	19 (1)
*Karnofsky performance score*	
60	4
70	12
80	5
90	3

Values are mean (s.e.m.) or total number of patients.

. Patients were elderly (mean age 68), and there was a preponderance of female subjects. Patients had an average BMI of 20 and were malnourished having lost, on average, 19% of their preillness stable weight. In general, the patients' Karnofsky score was moderately impaired, indicating that they were self-caring but unable to carry on normal activities or do active work.

Patients' baseline measured and predicted levels of REE, TEE, EEA and PAL are shown in Table 2

**Table 2:** Comparison of baseline measured and predicted REE, TEE, TEI and PAL of weight-losing patients with unresectable pancreatic cancer (*n*=24)

	**Measured**	**Predicted**	***P***
REE (kcal day^−1^)	1387 (42)	1268 (32)	0.001
TEE(kcal day^−1^)	1732 (82)	1903 (48)	0.023
TEI (kcal day^−1^)	1754 (95)	1903 (48)	0.007
EEA (kcal day^−1^)	345 (60)	634 (16)	0.001
PAL	1.24 (0.04)	1.50	—

Values are mean (s.e.m.), comparisons by Student's paired *t*-test.

. Patients had a significantly elevated REE when compared with predicted values. Conversely, their EEA and TEE were significantly lower than predicted. Patients' TEIs were significantly lower than predicted values, but were not significantly different from measured TEE.

Following randomisation of the patients willing to participate in this extended protocol of the larger trial, 15 patients were allocated to the control energy and protein dense oral nutritional supplement and nine patients to the n-3 enriched oral supplement. The mismatch in sample size was due to the larger study being stratified and the investigators being blinded until the study was complete. Patient characteristics at baseline according to treatment group are shown in Table 3

**Table 3:** Baseline characteristics of 24 weight-losing patients with unresectable pancreatic cancer randomised to either control or n-3 fatty acid containing (experimental) sip feed

	**Control (*n*=15)**	**Experimental (*n*=9)**	***P***
Sex (M : F)	4 : 11	6 : 3	0.068
Age (years)	70 (3)	65 (2)	0.159
*Stage of disease*			
II	8	7	0.237
III	3	1	
IV	4	1	

BMI (kg m^−2^)	20 (1)	21 (1)	0.961
% weight loss in previous 6 months	19 (2)	21 (2)	0.500
*Karnofsky performance score*			
60	3	1	0.871
70	6	6	
80	5	—	
90	1	2	

Values are mean (s.e.m.) or total number of patients; comparisons by Student's *t*-test, Fisher's exact or Wilcoxon's test where appropriate.

**Figure 1:**
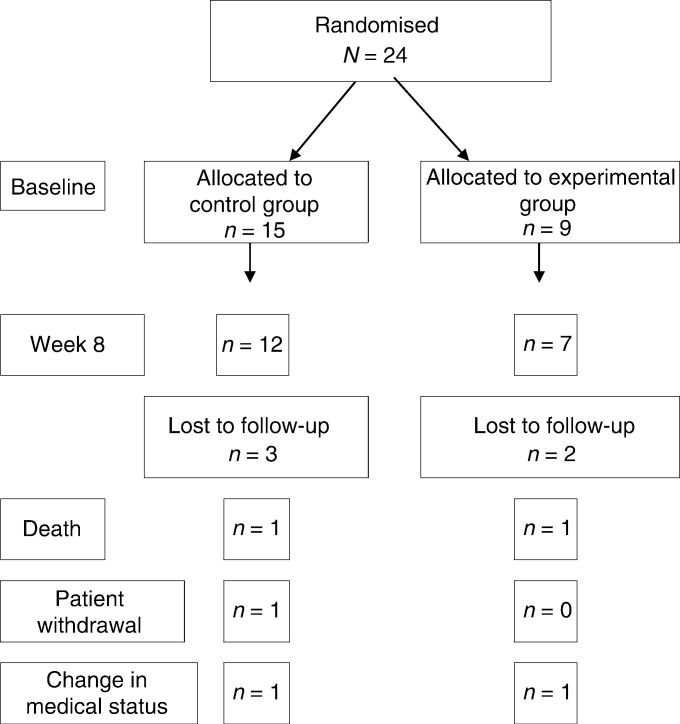
Pattern of randomisation and reasons for sample attrition.

. There were no significant differences between the groups. Five patients failed to complete the 8-week period of oral supplementation. The reasons for sample attrition are shown in Figure 1

.

The baseline meal intake, average oral supplement consumption and change in total dietary intake over the 8 weeks of the study are shown in Table 4

**Table 4:** Baseline meal intake, average oral nutritional supplement intake and change in total dietary intake for patients in the experimental and control groups reaching week 8

	**Control (*n*=12)**	**Experimental (*n*=7)**	***P***
Baseline energy intake (meals) (kcal day^−1^)	1814 (111)	1574 (178)	a
Baseline protein intake (meals) (g protein day^−1^)	73 (4)	57 (7)	a
Supplement energy intake (kcal day^−1^)	461 (56)	576 (44)	a
Supplement protein intake (g protein day^−1^)	24 (3)	30 (2)	a
Change in total energy intake^b^ (kcal day^−1^)	166 (122)	474 (106)	a, c, d
Change in total protein intake^b^ (g protein day^−1^)	4 (5)	27 (6)	c, d, e

^a^Control change *vs* experimental change, *P*: NS.

b That is, meals plus supplement intake during week 8 minus baseline meal intake (week 0).

^c^Control change from baseline, *P*: NS. ^d^Experimental change from baseline, *P*< 0.05. ^e^Control change *vs* experimental change, *P*< 0.05. ^f^Values are mean (s.e.m.); comparisons by Student's *t*-test for independent samples and comparisons within groups by paired Student's *t*-test.

. No patient reported intake of high n-3 fatty acid containing foods. The recommended supplement dose was 2 cans day^−1^. The mean intake of the control supplement was 1.5 cans day^−1^ and that of the n-3 fatty acid enriched supplement was 1.9 cans day^−1^ (*P*=0.126). An external review of randomisation procedures confirmed that all patients received the correct supplement. Compared with baseline intake, energy and protein intake increased significantly in those randomised to the n-3 enriched supplement. The total energy and protein intake did not increase significantly in those who received the isocaloric, isonitrogenous control supplement. Between-group comparisons revealed a trend towards increased change in TEI and a significantly greater increase in the total protein intake in those randomised to the n-3 enriched supplement.

Observed changes in weight, LBM, REE, TEE, EEA and PAL after 8 weeks of oral supplementation are shown in Table 5

**Table 5:** Change (i.e. week 8 minus week 0) in weight,LBM, REE, TEE, EEA and PAL for patients in the experimental and control groups reaching week 8

	**Control (*n*=12)**	**Experimental (*n*=7)**	***P***
Change in weight (kg)	−0.2 (0.8)	0.0 (1.3)	a,b,d
Change in LBM (kg)^e^	0.6 (0.8)	0.3 (0.5)	a,b,d
Change in REE (kcal day^−1^)	−15 (25)	−1 (42)	a,b,d
Change in TEE (kcal day^−1^)	99 (132)	286 (79)	a,c,d
Change in EEA (kcal day^−1^)	114 (131)	287 (66)	a,c,d
Change in PAL	0.01 (0.1)	0.18 (0.05)	a,c,d

Values are mean (s.e.m.) comparisons between groups by Student's *t*-test for independent samples and comparisons within groups by paired Student's *t*-test. ^a^ Control change from baseline, *P*: NS. ^b^ Experimental change from baseline, *P*: NS. ^c^ Experimental change from baseline, *P*<0.05. ^d^ Control change *vs* experimental change, *P*: NS.

e Changes in LBM calculated from TBW measurements using isotope dilution methodology.

. Compared with baseline values, there were no significant changes in weight or LBM in either group over the 8-week period of supplementation. When compared with baseline values, there were no significant changes in TEE, REE, EEA or PAL in patients randomised to the control supplement. In contrast, when compared with baseline values, TEE, EEA and PAL all increased significantly in those randomised to the n-3 enriched supplement. There were, however, no statistically significant differences between groups.The mean baseline and week 8 plasma EPA levels from patients in either group completing the 8-week period of oral supplement consumption are shown in Table 6

**Table 6:** Baseline and final plasma phospholipid EPA levels in patients in the experimental and control groups who completed 8-weeks oral supplementation

	**Control (*n*=12)**	**Experimental (*n*=7)**	***P***
Baseline EPA (%)	0.91 (0.21)	0.94 (0.09)	0.933
Patients (*n*) with baseline EPA>1.6%	2	0	—
Final EPA (%)	1.70 (0.49)	5.57 (0.70)	0.001
Patients (*n*) with final EPA>1.6%	4	7	—

Values are mean (s.e.m.) with comparisons between groups by Student's *t*-test for independent samples.

. At baseline, the levels of EPA were low and two control patients had levels above the 90th centile of 1.6% of unsupplemented pancreatic cancer patients (see Patients and Methods). After 8 weeks, the EPA levels of these two patients had risen further (2.8 and 3.1%) and two more of the 12 patients in the control supplement group had levels of EPA >1.6%, thereby suggesting consumption of EPA from a source outwith the trial, and indicting noncompliance with the protocol.

On the basis that four patients taking the control supplement had high EPA levels, a *post hoc* analysis based on plasma EPA levels was undertaken (Table 7

**Table 7:** Change (i.e. week 8 minus week 0) in REE, TEE, EEA and PAL for patients allocated in a *pos hoc* analysis to either the low or high EPA groups based on week 8 plasma phospholipid EPA levels

	**Low EPA group (*n*=8)**	**High EPA group (*n*=11)**	
Change in REE (kcal day^−1^)	−55 (23)	23 (30)	a,b,c
Change in TEE (kcal day^−1^)	−68 (134)	340 (92)	a,b.d
Change in EEA (kcal day^−1^)	−13 (143)	318 (93)	a,b,c
Change in PAL	0.01 (0.10)	0.24 (0.08)	a,b,c

Values are mean (s.e.m.); comparisons between groups by Student's *t*-test for independent samples and comparisons within groups by paired Student's *t*-test. ^a^ Control change from baseline: NS. ^b^ Experimental change from baseline: *P*<0.05. ^c^ Control change *vs* experimental change: NS. ^d^ Control change *vs* experimental change: *P*<0.05.

), in which these four patients were considered part of the high EPA group. This analysis revealed results similar to the ITT analysis. TEE, EEA, REE and PAL increased significantly from baseline in the ‘high EPA’ group, but not in those in the low EPA group. In addition, following 8-weeks supplementation change in TEE was significantly greater (*P*=0.018) and there was a trend towards higher EEA (*P*=0.06) and PAL (*P*=0.08) in the ‘high EPA’ group compared with the ‘low EPA’ patients.

## DISCUSSION

The present study demonstrates that in cachectic cancer patients, REE is increased but that TEE and therefore physical activity is reduced. These findings confirm previous studies, which have demonstrated that REE is increased in cachectic patients with pancreatic cancer (Falconer *et al*, 1994), and are consistent with numerous other studies, which have suggested that at least a proportion of weight-losing cancer patients are hypermetabolic (Dempsey *et al*, 1986; Fredrix *et al*, 1991). Such hypermetabolism is potentially of great significance to the weight-losing patient. First of all it may accelerate wasting by increasing any energy deficit already present due to anorexia. Second, as shown in the present study, it may be associated with a reduction in PAL. Such decreased physical activity may represent an adaptive response in which physical activity is reduced in an attempt to narrow the deficit between TEI and energy expenditure. Alternatively, it may reflect a negative effect on physical activity secondary to the patient's primary disease process or, indeed, a combination of the two.

A previous study using a urea-bicarbonate 36 h protocol in patients with lung cancer (Gibney *et al*, 1997) has documented a similar pattern of increased REE and decreased TEE and PAL. However, not all hypermetabolic patients have reduced physical activity. For example, Heijligenberg *et al* (1997) demonstrated relative hypermetabolism in a group of patients with HIV infection but observed relatively normal TEE and PAL. These patients were not wasted or losing weight at the time of study, and presumably were able to maintain energy balance by eating slightly more. This tends not to be the case in advanced cancer where anorexia and early satiety are often dominant symptoms.

The level of physical activity observed in the present study was markedly reduced (mean PAL: 1.24) compared with the value for healthy sedentary adults (PAL: 1.4–1.5; (Black *et al*, 1996). Such a PAL is comparable with that observed in spinal cord-injured patients living at home (mean PAL: 1.32; Mollinger *et al*, 1985) or patients with cerebral palsy (mean PAL: 1.23; Stallings *et al*, 1996). In the present study, the mean Karnofsky performance score of the patients was 70, suggesting that they were ‘self-caring but unable to carry on normal activities or do active work’. The data from the doubly labelled water studies would certainly confirm that patients were functioning at or below such a level. These findings attest to the marked impact of advanced cancer and cachexia on the physical function and quality of life of such patients.

In the present study, the patients' mean baseline calorie intake from food was 1754 kcal day^−1^. This represented less than what would be predicted for food intake had such individuals been healthy (1903 kcal day^−1^: see Table 2), but was roughly equivalent to measured TEE. From the recall of their preillness stable weight, the patients had lost approximately 20% of their normal weight, equivalent to about 2–3 kg month^−1^. However, if their energy intake matched their energy expenditure, this would suggest either that the patients' evident energy deficit (cf previous weight loss) had become attenuated or that the diet diary method used to measure food intake had systematically overestimated the patients' intake. Previous studies in obese patients have suggested a systematic bias towards underestimation of food intake in patients undertaking dietary restriction (Lissner *et al*, 1998). The findings of the present study caution against the overinterpretation of diet diary data in studies where cachectic patients may want to please their physician or relatives by systematically overestimating their food intake.

Following baseline assessment, patients were randomised to receive for 8 weeks an energy and protein dense oral supplement with or without n-3 fatty acids. During the 8 weeks, the patients' weight and LBM remained stable. However, the net increase in both protein and energy intake in the patients taking the n-3 enriched supplement (Table 4) seemed to translate into an almost parallel increase in TEE with a consequent rise in PAL. In contrast, those receiving the isocaloric, isonitrogenous control supplement had no significant increase in net intake and no change from baseline in terms of TEE or PAL. These data are unique in documenting (with objective methodology) an improvement in the physical function of cachectic patients with advanced cancer following institution of combination therapy (i.e. n-3 fatty acids (EPA) and energy and protein dense oral supplements). A previous study with this combination regimen (Barber *et al*, 1999) in patients with pancreatic cancer demonstrated a similar net rise in energy intake (400 kcal day^−1^) with no change in fat mass but a significant improvement in performance status (Karnofsky score). These findings parallel those of the present study with translation of extra energy intake into increased physical activity rather than energy storage. The mechanism for this effect remains unclear but may relate to the action of EPA on various mediator pathways (e.g. cytokines or PIF).

In a previous study with the same experimental supplement in similar patients (Barber *et al*, 1999), LBM increased significantly (2 kg in 7 weeks). In the present study, patients' LBM did not change significantly. In the overall randomised trial (of which the present study was a part), compliance with the experimental supplement averaged 1.4 cans day^−1^ (Fearon *et al*, 2003), which was less than in the study by Barber *et al* (1999) or in the present substudy (both 1.9 cans day^−1^). Equally, LBM did not change significantly in the overall trial although there was a positive, direct, significant relationship between supplement intake and change in LBM in the experimental group but not in the control (Fearon *et al*, 2003). It is therefore not clear whether the increased TEE and PAL documented in the present study is necessarily accompanied by an increase in the mass rather than in the function/quality of the LBM. This observation raises the issue of whether trials in the treatment of cancer cachexia should focus exclusively on nutritional rather than functional end points.

In the present study, analysis of the patients' plasma EPA at week 8 revealed strong evidence that some of the control subjects had been taking an exogenous source of EPA. When the results were reanalysed on the basis of plasma EPA levels, the same trends observed in the ITT analysis were observed and some became more obvious. However, such *post hoc* analysis has to be treated with caution and will require confirmation in further studies. The numbers of patients included in the present study was relatively small and fell slightly short of the intended recruitment. Analysis was further hampered by the randomisation process allocating more patients to the control group compared with the experimental group. Nevertheless, the present study provides pilot data, which suggest that in contrast to an isocaloric, isonitrogenous control nutritional supplement, administration of a supplement enriched with n-3 fatty acids results in an increase in PAL. Further work is required to confirm these results and clarify the relationship between PAL and overall quality of life. Documentation of TEE and PAL using the DLW technique is labour intensive and costly. However, the data obtained are unique and highlight the potential of such objective methodology to provide a focus for therapeutic intervention in the complex syndrome of cancer cachexia.
